# Effect of platelet-rich plasma on polypropylene meshes implanted in the rabbit vagina: histological analysis

**DOI:** 10.1590/S1677-5538.IBJU.2016.0177

**Published:** 2017

**Authors:** Natália Gomes Parizzi, Oscar Ávila Rubini, Silvio Henrique Maia de Almeida, Lais Caetano Ireno, Roger Mitio Tashiro, Victor Hugo Tolotto de Carvalho

**Affiliations:** 1 Departamento de Cirurgia, Universidade Estadual de Londrina, Londrina, PR, Brasil;; 2 Departamento de Cirurgia, Universidade do Oeste Paulista, Presidente Prudente, SP, Brasil

**Keywords:** Platelet-Rich Plasma, Collagen, Rabbits, Inflammation

## Abstract

**Purpose:**

The polypropylene mesh (PPM) is used in many surgical interventions because of its good incorporation and accessibility. However, potential mesh-related complications are common. Platelet-rich plasma (PRP) improves the healing of wounds and is inexpensive. Thus, the purpose of this study was to analyze the effect of the PRP-gel coating of a PPM on inflammation, production of collagen, and smooth muscle in the rabbit vagina.

**Materials and Methods:**

The intervention consisted of a 1.5cm incision and divulsion of the vaginal mucosa for the implantation of a PRP-coated PPM. The PRP-coated mesh was implanted in 15 rabbits, and in the second group, the same implant was used without the PRP coating. In the sham group, the intervention consisted of the incision, divulsion, and suture. The rabbits were euthanized at 7, 30 and 90 days, and full-thickness sagittal sections of the posterior vaginal wall and rectum were scored. The inflammatory infiltrate was evaluated using hematoxylin and eosin staining. The Sirius Red stain was used to examine deposition of collagen I and III, and Masson’s trichrome staining was used to visualize the smooth muscle.

**Results:**

The group with PRP-coated meshes had a lower inflammatory infiltrate count at 30 days. Deposition of collagen III increased with the use of PRP-coating at 90 days.

**Conclusions:**

The area of inflammatory infiltrate was significantly increased in the group without the PRP-coated mesh at 30 days but not in the group with the PRP-coated mesh, indicating a less intense inflammatory response. In addition, a significant increase in collagen III occurred at 90 days.

## INTRODUCTION

The integration between meshes and vaginal tissue depends on the structure of the mesh and on factors such as tissue tropism, infection, and inflammation; these factors are also directly related to the risk of complications (1-4).

Rechberger et al. observed that the serum levels of cytokines are higher in patients with erosion slings than in those with proper healing (5). Similarly, Di Vita et al. demonstrated that the use of polypropylene for hernia repair is associated with high levels of interleukin-6 and interferon, when compared to traditional correction (6). Thus, chronic inflammation with a large foreign body reaction promotes the occurrence of complications.

Previous studies have evaluated coating meshes with materials known for their potential to accelerate healing and attenuate the inflammatory response and downstream fibrosis and to modulate collagen deposition (7, 8). The composition of these coatings varies from synthetic materials including prophylactic antimicrobials and metal films to biologic materials such as collagen (7-10). Unfortunately, the results are inconsistent.

However, there are few studies on the use of PRP on meshes for the repair of hernias and in Urogynecology. An in vitro study performed using seven types of meshes with PRP showed a reduction in adhesions and improved biocompatibility after 6 weeks (11). In addition, in two experiments, using PRP in a biological loop resulted in less severe adhesions, increased angiogenesis, increased neovascularization, increased integrity of the fabric, and a decrease in the recurrence of hernia in the group with PRP (12).

Similarly, Gerullis et al. conducted an in vitro study comparing polypropylene-coated meshes coated with peripheral blood mononuclear cells, platelets, and plasma. They concluded that the autologous plasma promoted increased biocompatibility of fabrics, justifying in vivo studies (11).

Smooth muscle is often a minor contributor to resisting passive mechanical loading; however, it is extremely important in maintaining vaginal tone and actively resisting the forces of the surrounding connective tissues. Few studies have thoroughly investigated the impact of synthetic meshes on the smooth muscle, also known as functional properties (13).

To improve the understanding of the impact of meshes on the vagina, multiple mechanisms that could affect the properties of the vaginal tissue, like smooth muscle, should be investigated.

The PRP is acquired by centrifuging plasma in order to obtain a platelet and leukocyte concentration 2 to 3 times higher (on average) than the regular plasma and is a clinical option for accelerating the healing process in hernia correction with meshes (14, 15). PRP is easy to obtain at a low cost. Hence, we proposed coating the monofilament polypropylene mesh with PRP to study its effect on the inflammation process and collagen deposition in the vaginas of rabbits.

## MATERIALS AND METHODS

The sample consisted of 45 sexually mature, pure-bred, female, white rabbits aged 40 weeks and weighing 4.5kg. A pilot project was performed to test the methods and define the sample numbers. Three groups of fifteen rabbits were randomly generated: sham, vaginal deployment of 1.0cm polypropylene mesh with pores of 1500µm, and the same mesh coated with PRP gel obtained after removal of a sample of 10mL of blood obtained by cardiac puncture (a large blood volume proportional to the animal’s weight).

The blood sample was obtained during the implant procedure and was immediately taken to the laboratory for preparation using the same protocol as used for human samples (16). The specimens were anesthetized, and the blood was transferred to a sterile 1.8mL tube containing 0:10mL of citrate as an anticoagulant. The material was homogenized and centrifuged at 24ºC for 10 minutes. After centrifugation, it was possible to distinguish two distinct layers in the tube: the red blood cells at the bottom and the supernatant plasma. All plasma was removed with a pipette and placed in a sterile plastic tube. Additional centrifugation was performed at a speed of 1500rpm at 24°C. After centrifugation, the plasma around the top of the tube was removed, leaving only the portion to which 0.5mL 10% calcium gluconate was added*.* The solution was homogenized and allowed to stand for 30 minutes, acquiring a gel-like consistency.

Platelet counts were performed in 25% of the plasma samples chosen randomly before and after PRP preparation to confirm the increase in the number of platelets. The gel contained, on average, three times the platelet count of the peripheral blood. A 1–1.5cm vaginal incision was performed, and the implant was inserted, without fixation, to prevent tissue reactions. The mesh was inserted, in a standardized manner, between the vaginal epithelium and the rectovaginal fascia (17), and was coated with PRP gel so that the entire length and the interstice between the mesh pore was filled. The vaginal incision was closed with Vicryl. Penicillin was administered. The sham group underwent an operation consisting of the same vaginal incision using the same protocol.

The animals were divided into three groups of fifteen animals per group and 5 were euthanized at 7, 30 and 90 days after implantation. All were anesthetized before lethal injection. The implant site was removed en bloc*,* including the vagina, mesh, and rectum. At each time point, the wounds were harvested and their histologic features were assessed in paraffin-embedded sections using hematoxylin and eosin staining. The Sirius Red stain was performed and samples were assessed using polarized light microscopy, a simple, sensitive, and specific method for quantification of collagen. It is particularly useful for examining the heterogeneity of collagen fibers in connective tissues, providing essential information in pathological studies (18).

One pathologist, who was blinded to the tissue type and time from wounding, evaluated all specimens. The slides were scanned under a microscope. Collagen I and III were assessed using polarized light, by density per micra. The inflammatory infiltrates (INI) and muscle tissue (micra^2^) were counted in different fields. Four fragments of the material were placed on each slide.

All statistical analyses were carried out using the SPSS 20.0 system. We analyzed the hypothesis of normal distribution and homogeneity using the Shapiro and Levene tests. Because of a violation of normality, the data were analyzed using the Kruskal-Wallis test, followed by the Bonferroni test for comparisons between groups with and without PRP-coating and for comparisons between different time-points.

## RESULTS

An extrusion of polypropylene mesh occurred in each of the groups (with and without PRP-coating); these animals were excluded from the study and replaced. None of the animals died during the observation period. Moreover, none of them presented signs of systemic compromise or procedure-related complications.

Table 1 shows the results (median and interquartile range) at the euthanasia times.

**Tabela 1:** The median and the interquartile range at the euthanasia times, groups with and without PRP. Quantification by cells number (INI) and micra2 (collagen type I, type III and smooth muscle).

	Seven days		p Value
	**Without PRP**	**With PRP**	
**INI**	5.00 (1.00)	3.00 (3.00)	0.09
**Ci**	2874,01 (2140,42)	3455,19 (1040,62)	0.40
**CIII**	3060,48 (1094,56)	2398,66 (194,05)	0.26
**Muscle**	15522,15 (11707.01)	16829,06 (3085,59)	0.49

	30 Days		

**INI**	**141,00 (173,00) #**	**4,00 (0,00) #**	**0.0175#**
**Ci**	3463,84 (1836,11)	3846,56 (1614,01)	0.34
**CIII**	2613,64 (4687,18)	2543,25 (495,76)	0.17
**Muscle**	10216,80 (2361,56)	15085,41 (8758,95)	0.08

	90 days		

**INI**	20,00 (12,00)	19,00 (7,00)	0.6
**C III**	**2304,46 (1383,01) #**	**8617,72 (16671,74) #**	**0.022#**
**CI**	2247,62 (487,07)	1153,37 (18101,06)	0.098
**Musc**	6198,46 (1562,64)	10734,65 (9259,69)	0.061

**INI** = Inflammatory infiltrate; **C III** = Colagen III; **C I** = Colagen I; **musc** = muscle

#*P*<0.05 without PRP x with PRP Mann-Whitney test;

*P<0.05: Comparing days; Kruskall-Walis Test.

**Figure 1:**
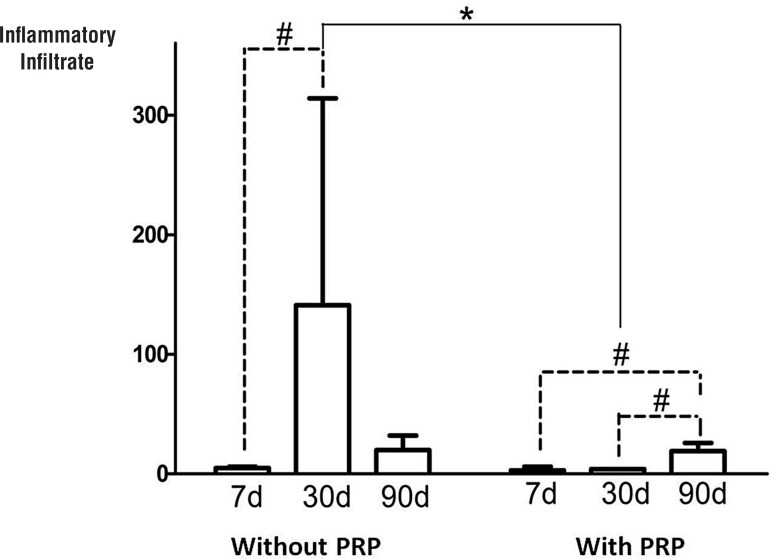
Inflammatory infiltrate (INI) area at different time points in groups with and without PRP-coating.

The amount of the inflammatory cells in the first seven days did not become elevated. However, at 30 days, the PRP-coated group had significantly lower levels of inflammatory cells than the group without PRP-coating (Figure-1). After 90 days, the inflammatory response between study groups was indistinguishable. The sham group had significantly lower levels of inflammatory cells at 30 and 90 days than the other groups.

*p<0.05 without PRP-coating versus with PRP-coating at 30 days (Mann-)

#p<0.05 without PRP-coating, comparisons between 7 and 30 days, Group with PRP-coating: comparisons between 7 and 30 days and 7 and 90 days (Kruskal)

**Figure 2:**
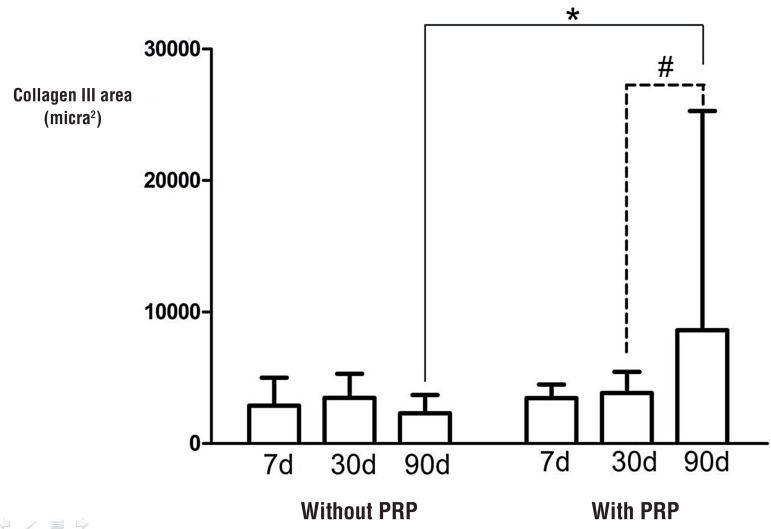
Comparisons between the median collagen III areas in rabbits sacrificed at 7, 30 and 90 days.

In the group without PRP-coating, the concentration of collagen III did not vary between euthanasia times. In the group with PRP-coating, this value was significantly increased at 90 days (Figure-2).

**Figure 3:**
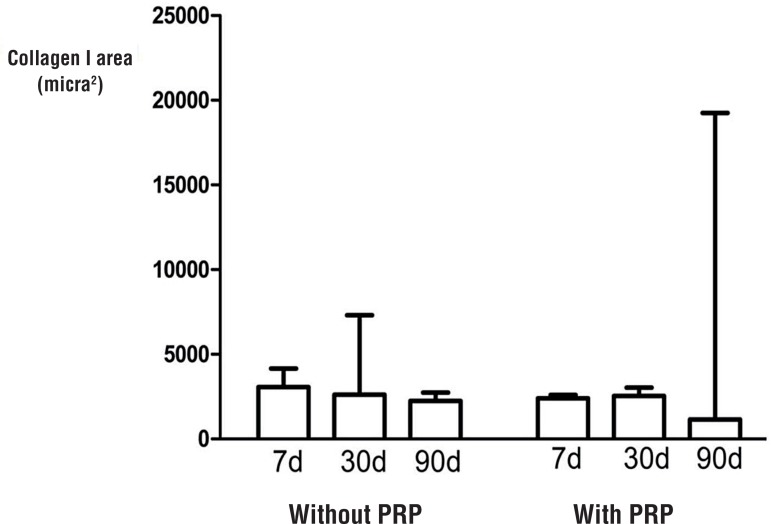
Comparisons of the median of collagen I area for the rabbits sacrificed at 7, 30 and 90 days.

**Figure 4:**
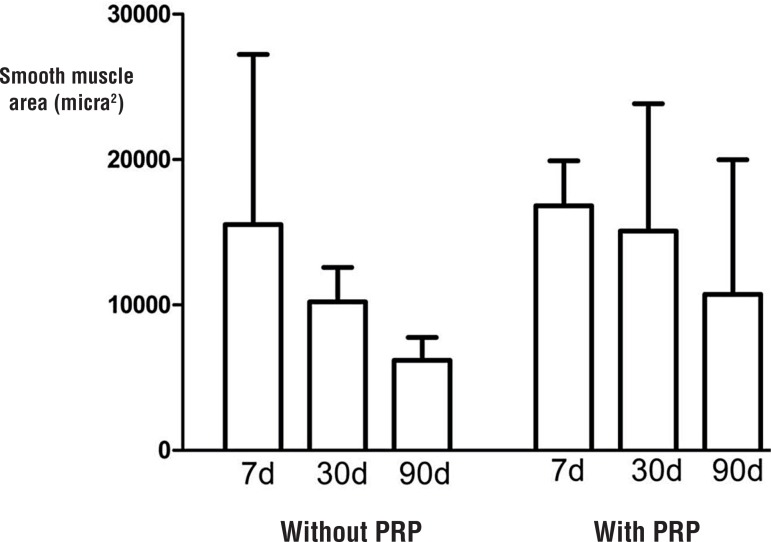
Comparisons of the median of smooth muscle area for the rabbits sacrificed at 7, 30 and 90 days.

The collagen I concentration did not vary with time and presence of PRP (Figure-3). The smooth muscle area showed a small increase; however, this was not significant (Figure-4).

**PRP** = Platelet-rich plasma.

**PRP** = Platelet-rich plasma.

## DISCUSSION

To our knowledge, this is the first study coating meshes with PRP for vaginal implants. The local inflammatory reaction is an early event that occurs after mesh implantation, and a subsequent foreign body reaction caused by the implant was already established after 3 months and did not significantly change over a 24-month period (11).

In the present study, an acute inflammatory reaction occurred in rabbits implanted with meshes both with and without PRP-coating after seven days, suggesting that the use of PRP did not affect this initial inflammatory process. At 30 days, the PRP-coated group showed a significant reduction in inflammatory cells, suggesting that the PRP-coating shortened the time of the acute inflammatory response, leading to an early tissue repair proliferative phase.

In both in vitro and in vivo studies, Gerullis et al. also noted that the use of plasma on various materials did not influence the early inflammatory reaction (11, 19). However, three months after implantation, markers of tissue vascularization organization (invasion of myofibroblasts and endothelial cells) were detectable and there were differences between the three meshes investigated.

In addition, in the PRP-coated group, at 90 days post-implantation there was a significant increase in type III collagen fibers, the first to be produced in the presence of inflammatory cells. The higher presence of immature collagen (III) in the PRP-coated group at 90 days suggests that the wound was in the phase characterized by Schultz et al. as contraction and remodeling (20). It has been shown that premature type III collagen is predominantly synthesized in the early phases of wound healing and in the presence of inflammatory cells. Collagen III is then replaced by highly cross-linked and stable collagen type I later after implantation, and this slowly increases the tissue tensile strength.

Remodeling of the extracellular matrix is essential for implant integration, and the mesh-induced foreign body responses must be balanced to result in normal wound healing. Swift and adequate tissue ingrowth into the mesh results in superior biocompatibility and likely improves the clinical performance. Intense or prolonged inflammation and bad infiltration, resulting in scar plate formation, can be accompanied by shrinkage or deformation of the biomaterial, recurrence, adhesion, fistula, or erosion of nearby tissue (21).

The vagina is comprised of both passive (collagen) and functional (smooth muscle) components. To date, studies have thoroughly investigated the impact of synthetic meshes on the active properties of the vagina. Tissue degeneration was found to be in large part related to mesh stiffness (22). Liang et al. showed that following implantation with a stiffer mesh, the vagina demonstrated evidence of a maladaptive remodeling response (23). This is characterized by the thinning of the smooth muscle layer, increased cell apoptosis, increased collagenase activity, decreased collagen and elastin content, and increased glycosaminoglycan content. Furthermore, Jallah et al. observed that the mesh has an overall negative impact on vaginal smooth muscle function. In this study, unfortunately, the PRP-coating did not change the size of the vaginal smooth muscle area (13).

As with any animal research, the extrapolation of the results to clinical practice should be carefully considered, but the vaginal implant model is an advantage of this study (24). The inflammatory response and repair go far beyond the type of mesh deployed; each tissue and implant site responds differently to aggression. Abdominal implants, for example, are placed in a sterile environment, with vastly different biomechanics than the vaginal implants, which are put into a potentially contaminated environment (25, 26). Thus, the use of implants in rabbits and vaginal mucosa are important points in our study.

The rabbit is considered a useful animal model for vaginal implants but is not a large primate mode. The rabbit’s vagina has two portions; the inner is more akin to small intestinal histology, but the wider segment of the external vaginal wall makes a suitable model for histocompatibility studies (26).

A possible limitation in this study is the age of the rabbits; all were of reproductive age and had a good vaginal trophism. It is known that hipoestrogenic vaginal mucosa is less receptive to implantation meshes, increasing the rates of complications (27). Likewise, postoperative estrogen replacement for eight weeks in rabbits increased collagen deposition in the vaginal mesh implantation (28). However, there is data that suggests that the PRP would have an even more positive action in this type of animal. Abramov et al. observed in spayed rabbits that collagen production is diminished in the healing of the vaginal mucosa and there is increased inflammation (29).

Another limitation of this study was that only one mesh type was investigated. We chose a monofilament and macroporous polypropylene mesh because it is the most accepted design based on the literature and is used in surgeries (30). A study about the different structural weights and pore sizes is one possible continuation of this research. It is also necessary for better understanding the action of PRP on the enzymatic and immunological processes involved in mesh integration.

Moreover, before clinical implementation, it is necessary to conduct further studies evaluating the use of PRP-coating on mesh implants in vaginas of oophorectomized, older, and multiparous animals.

## CONCLUSIONS

The inflammatory infiltrate area did not elevate in the group with platelet-rich plasma, at 30 days, indicating a less intense inflammatory response. Also, a significant increase of collagen type III occurred at 90 days of the study in the group with platelet-rich plasma.
